# Reviewers for *Journal of Insect Science* (November 2020–October 2021)

**DOI:** 10.1093/jisesa/ieac007

**Published:** 2022-05-04

The Editor-in-Chief and the Subject Editors of the *Journal of Insect Science* thank the following scientists for their voluntary commitment of valuable professional time and expertise to peer reviewing manuscripts submitted for publication in our journal. The quality and scientific stature of the journal depends on the conscientious efforts of these individuals.

## Top Reviewers (3+ reviews completed)

Gernat, Tim

Harpur, Brock

Hernandez, Emilio

Liao, Ling-Hsiu

Rinkevich, Frank

Sarfati, Raphaël

Srinivasan, Rajagopalbabu

**Reviewers**

Adamaki, Christina

Alburaki, Mohamed

Almeida, José Rafael De

Alto, Barry

Andow, David

Andric, Goran

Araujo, Douglas

Aristizabal, Luis

Attardo, Geoffrey

Avalos, A.

Backus, Elaine

Bakhsh, Allah

Baliota, Georgia

Bandani, Ali

Bansal, Raman

Barbercheck, Mary

Barribeau, Seth

Bartels, David

Bartlett, Lewis

Battisti, Andrea

Bernadou, Abel

Bernal, Julio

Bernaola, Lina

Blaauw, Brett

Bonte, Jochem

Bouvet, Juan Pedro

Brent, Colin

Bruninga-Socolar, Bethanne

Buchman, Anna

Burnett, Nicholas

Buttstedt, Anja

Cai, Pumo

Cameron, Stephen

Castillo, Alfredo

Castro, Ruann Janser Soares De

Celorio-Mancera, Maria De La Paz

Cervantes, Felix

Cha, Dong H.

Chakrabarti, Priyadarshini

Chaudhury, Muhammad

Chen, Jiahua

Chen, Maohua

Chen, Xue-Xin

Clemes Cardoso, Danon

Cloonan, Kevin

Colla, Sheila

Cook, Don

Dai, Fangyin

Danihlik, Jiri

Daugherty, Matt

De Lange, Elvira

Delabie, Jacques Hubert Charles

Delaplane, Keith

Dharavath, Neethu

Diaz-Fleischer, Francisco

Dietrich, Christopher

Dingha, Beatrice

Do Nascimento, Antonio

Donoso, David

Dorrah, Moataza

Duffield, Kristin

Dumonceaux, Tim J.

Durant, Jennie

Dutra, Heverton

Edwards, Owain

Ellis, James

Erler, Julius

Evans, Jay

Faiman, Roy

Falabella, Patrizia

Fiedler, Konrad

Finch, Elizabeth

Firlej, Annabelle

Fisher, Adrian

Flenniken, Michelle

Fragoso, Fabiana

Freitak, Dalial

Fruttero, Leonardo L.

Furukawa, S

Galante, Eduardo

Geden, Chris

Giacomini, Jonathan

Goñi, Beatriz

Gonzã¡Lez, Ezequiel

Gorman, Maureen

Gourgouta, Marina

Guedes, Raul Narciso

Guedot, Christelle

Guzmán-Franco, Ariel

Guzman-Novoa, Ernesto

Haddi, Khalid

Hagler, James

Halbert, Susan

Harrison, Jon

Hastings, John

Hartfelder, Klaus H.

Hellmich, Richard

Hendry, Tory

Hervet, Vincent

Hesler, Louis

Hidalgo, Sergio

Hilliou, Frédérique

Hines, Heather

Hinkelman, Travis M.

Hoffmann, Ary

Hou, Chunsheng

Huang, Juan

Hull, Joe

Huston, Daniel

Jack, Cameron

Jaffe, Benjamin

Jaumann, Sarah

Jiao, Xiaoguo

Johnson, Reed

Joshi, Rakesh

Joubert, Albert

Jung, Chuleui

Kainoh, Yooichi

Kavanagh, Kevin

Keena, Melody

Kellermann, Vanessa

Kendall, Liam

Keshan, Bela

Kesheimer, Katelyn

Kohl, Patrick

Kondur, Yalçın

Kreuzer, Michael

Krishnan, Natraj

Kuhar, Thomas

Kulma, Martin

Lasa, Rodrigo

Latorre-Estivalis, Jose Manuel

Lattorff, Michael

Le Goff, Gaelle

Leach, Heather

Lee, Jana

Lehmann, Philipp

Leitao, Alex

Lemic, Darija

Lesne, Pierre

Lester, Phil

Li, Zhilin

Liu, Jian

Liu, Jisheng

Liu, Pengcheng

Liu, Xianhui

Liu, Xingyue

Liu, Yan-Qun

Lourenco, Anete

Ma, Tao

Ma, Weihua

Malaker, Stacy

Maluta, Nathalie Kristine Prado

Malysh, Julia M.

Manfredini, Fabio

Manteca-Acosta, Mariana

Martinez, Julien

Matsumoto, Yoshiki

Mayfield, Albert

Mcafee, Alison

Mcculloch, Graham

Meagher, Robert

Meloni, Fernando

Merzendorfer, Hans

Metz, Bradley

Miller, Nicholas

Minahan, Danny

Mishra, Ruchir

Mizuno, Takafumi

Mohamed, Amr

Mola, John

Montoya, Pablo

Mori, Boyd

Moura, Mariana

Muth, Felicity

Muttis, Evangelina

Nair, Parvathi

Naorem, Anjana

Neff, Felix

Nguyen, Chuong V.

Nikolouli, Katerina

Nixon, Laura

Obregon, Diana

Oliveira, Paulo

Oliver, Shune

Otti, Oliver

Papanikolaou, Nikos

Pascacio, Carlos

Patch, Harland

Pehlivan, Serkan

Pereira, Adriano

Pickett, Charles

Pietri, Jose

Pinero, F. S.

Pinero, Jaime

Portilla, Maribel

Prazic Golic, Marijana

Rakhshani, Ehsan

Ramirez-Cruz, A

Ramos, Letícia Fernanda

Ranger, Christopher

Ricupero, Michele

Riddick, Eric

Riggi, Laura

Rivera-Vega, Loren J.

Rodrigues, Sergio Roberto

Rodriguez-Saona, Cesar

Romanholo Ferreira, Luiz Fernando

Romano, Donato

Rossi, Natacha

Rueppell, Olav

Rugman-Jones, Paul

Rumbos, Christos

Sadd, Ben

Sagili, Ramesh

Sandanayaka, W. R. M.

Sato, Takuya

Scannapieco, Alejandra C.

Scott, Maxwell

Sen, Sandeep

Sheldon, Fran

Shrewsbury, Paula

Silva, Diego

Silva, Luciana

Simo, Ladislav

Smagghe, Guy

Smith, Brian

Song, Jiasheng

Spivak, Marla

Srivastava, Vicek

Staab, Michael

Sterkel, Marcos

Sugiura, Shinji

Suma, Pompeo

Swevers, Luc

Tahir, Hafiz Muhamamd

Takemoto, Hiroyuki

Takiya, Daniela

Tarpy, David

Tison, Lea

Torma, Attila

Traynor, Kirsten

Turchen, Leonardo

Ullah, Muhammad

Ulyshen, Michael

Underwood, Robyn

Urban, Julie

Urbaneja-Bernat, Pablo

Usseglio-Polatera, Philippe

Vandenbrooks, John M.

Vandervoet, Timothy

Vargas, German

Vavassori, Laura

Victoria, Soroker

Villagómez, Gemma

Villanassery Joseph, Shimat

Wagner, Anika E.

Wagoner, Kaira

Walsh, Elizabeth

Wang, Hualing

Wang, Luoluo

Wang, Xiao-Yi

Weintraub, Phyllis

Weiss, Brian

Wen, Xiujun

Williams, Trevor

Wolt, Jeff

Woodgate, Joseph

Wu, Hua

Xu, Li

Zahniser, James

Zalucki, Myron

Zengin, Erdal

Zhang, Wei

Zhu, Gengping

